# Ecological factors and parity mode correlate with genome size variation in squamate reptiles

**DOI:** 10.1186/s12862-023-02180-4

**Published:** 2023-12-05

**Authors:** Anik Saha, Arianna Bellucci, Sara Fratini, Stefano Cannicci, Claudio Ciofi, Alessio Iannucci

**Affiliations:** 1 Department of Biology, University of Florence, Sesto Fiorentino, Italy; 2 National Biodiversity Future Center, 90133 Palermo, Italy

**Keywords:** *C*-value, Ecology, Reproduction, Life history, Lizards, Snakes, Vertebrates, Microhabitat, Viviparity, Oviparity

## Abstract

**Background:**

Evidence of correlation between genome size, the nuclear haploid DNA content of a cell, environmental factors and life-history traits have been reported in many animal species. Genome size, however, spans over three orders of magnitude across taxa and such a correlation does not seem to follow a universal pattern. In squamate reptiles, the second most species-rich order of vertebrates, there are currently no studies investigating drivers of genome size variability. We run a series of phylogenetic generalized least-squares models on 227 species of squamates to test for possible relationships between genome size and ecological factors including latitudinal distribution, bioclimatic variables and microhabitat use. We also tested whether genome size variation can be associated with parity mode, a highly variable life history trait in this order of reptiles.

**Results:**

The best-fitting model showed that the interaction between microhabitat use and parity mode mainly accounted for genome size variation. Larger genome sizes were found in live-bearing species that live in rock/sand ecosystems and in egg-laying arboreal taxa. On the other hand, smaller genomes were found in fossorial live-bearing species.

**Conclusions:**

Environmental factors and species parity mode appear to be among the main parameters explaining genome size variation in squamates. Our results suggest that genome size may favour adaptation of some species to certain environments or could otherwise result from the interaction between environmental factors and parity mode. Integration of genome size and genome sequencing data could help understand the role of differential genome content in the evolutionary process of genome size variation in squamates.

**Supplementary Information:**

The online version contains supplementary material available at 10.1186/s12862-023-02180-4.

## Introduction

Genome size, the nuclear haploid DNA content of a cell, spans over three orders of magnitude across animals [1, 2]. Patterns of correlations between genome size and cytological traits are well known for a variety of taxa. For instance, a positive correlation exists between genome size and nucleus/cell size and a negative relationship was recorded between genome size and cellular division rate [3, 4]. Likewise, evidence of correlation between genome size and environmental factors and life-history traits such as temperature, salinity, relative humidity and developmental mode were found to influence genome sizes in both animal and plant species (e.g., [5–11]). However, the correlation between ecological factors and genome size does not seem to follow a universal trend and the degree of correlation often differs among taxa. For this reason, studies investigating variability among phylogenetically close species (e.g., same order or family) may help describe how life history and the environment relate to genome size evolution.

Extant squamates constitute one of the most species-rich order of vertebrates with over 11,000 described species. They include all lizards and snakes and are characterized by a great diversity of forms as well as physiological and ecological adaptations [12]. Squamates are found on every continent except Antarctica and have evolved under a variety of climatic conditions [13, 14]. Past climate changes had a particularly strong influence on the biology of ectotherm species with respect to endotherms [15] and climatic niche constraints may have played a key role in the evolution of genome size.

In fishes, freshwater and eurybiotic species have larger genomes than marine and stenobiotic taxa, respectively [6, 11, 16]. In marine fishes, genome size appears to increase with sea depth [16, 17]. Similar results were recorded for crustaceans in which large genome sizes characterized freshwater and deep-sea species [8, 18]. An association between genome size and ecological factors and life history traits was also suggested in amphibians [9, 10]. Microclimatic factors such as temperature and humidity in particular were found to affect genome size indirectly, as a consequence of their effect on reproduction and developmental modes [10].

For squamate reptiles, there are currently no studies on the correlation between genome size and ecological parameters. Chen et al. [19] investigated whether genome size variation was correlated with life history traits including clutch size, hatching time, hatchling mass and body mass in 199 species and found a correlation between genome size and clutch size only. No ecological factors were considered. In this study, we tested for correlation between genome size and ecological factors in 227 species of squamates. We considered latitudinal distribution, macroclimatic data and microhabitat use. Parity mode is a well conserved life history trait in all vertebrate groups except squamates, where it shows a high level of variability. We also tested whether genome size was associated in particular with live-bearing and egg-laying modes [20, 21].

## Materials and methods

### Genome size data

Haploid DNA contents per nucleus (*C*-values) were obtained for 227 species of squamates from the Animal Genome Size Database [2] and the GoaT database [22]. Analysis methods for each species are reported in the Additional file 1. The genome size dataset included 29 out of 72 families described for squamates [12]. An average *C*-value was calculated for species with multiple genome size records which differed by less than 2 pg in DNA content per nucleus.

### Ecological data

Global occurrence of the 227 species of squamates was retrieved from the Global Biodiversity Information Facility (GBIF, www.gbif.org) using the occ_search function of the rgbif package v3.7.2 [23]. The function clean_coordinates of the CoordinateCleaner v2.0–20 package [24] was used to remove records with no latitude and longitude values, ambiguous reports of occurrence at sea of terrestrial species, museum samples and species locations recorded outside the country of origin. An additional filtering step was performed using the dplyr package v1.0.8 [25] to remove locations with coordinate accuracy lower than 100 km, fossil records and occurrence with individual count values not parsable into a positive integer. Latitude and longitude median values were then calculated following Rotenberry and Balasubramaniam [26]. The median latitude absolute value was used for statistical analyses.

Macro-climatic data were obtained from 19 bioclimatic variables (Bioclim1–19) available from the WorldClim database v2.1 (www.worldclim.org) at a spatial resolution of 2.5 arc minutes using the raster function of the raster package v3.5–15 [27]. The median values of the 19 variables were assessed for each grid cell of a species’ location using the function SpatialPoints and the subroutine coordinates implemented in the package sp. v1.4–6 [28].

Microhabitat information for each species was retrieved from the available literature (See Additional file 1) and classified according to Bars-Closel et al. [29]. Taxa were distinguished among aquatic (active in marine and/or freshwater environment), arboreal (commonly found on trees), fossorial (living in burrows or under the leaf litter), rock/sand dwellers (living in deserts, cliffs or dry environments) and terrestrial (active on the ground) species.

### Parity mode data

Parity mode data were retrieved from The Reptile Database as three different categories [12]: oviparity (embryos that develop outside the mother’s body and absorb nutrients from the egg yolk), viviparity (embryos developing inside the mother’s body and receive nutrients directly from the mother) and ovoviviparity (embryos that develop either completely or partially inside the mother’s body but absorbs nutrients mainly from the egg yolk). Given that for a number of taxa it is difficult to draw a line between viviparity and ovoviviparity, many authors prefer to distinguish between egg-laying (oviparous) and live-bearing (viviparous and ovoviviparous) species (e.g., [30–34]). In this study, we pooled viviparous and ovoviviparous species in a single category and classified species as either egg-laying or live-bearing.

### Statistical analyses and phylogenetic correction

Phylogenetic relationships were based on Zheng and Wiens [35]. The original nexus tree was pruned to include only the 227 species of our dataset using the drop.tip function of the ape v5.7-1 package  [36]. We performed a phylogenetically corrected Principal Component Analysis (pPCA) based on the covariance matrix of the means of intraspecific bioclimatic variables Bio1–19 to reduce the dimensionality of the macroclimatic dataset using the phyl.pca function of phytools v1.0–1 [37, 38]. We implemented a pPCA instead of a standard PCA to avoid bias in model fitting caused by the non-independence of species-specific evolutionary histories [39–41]. The values of bioclimatic variables were log-transformed prior to the pPCA and then transformed into z-scores (describing the position of a raw score in terms of its distance from the mean when measured in standard deviation units) to account for variation in the measurement units among variables. We followed the broken-stick criterion [42] to retain those PC axes which explained the largest variation among climatic niches.

Phylogenetic correlations for discrete (microhabitat and parity mode) and continuous (*C*-value and latitude) variables were measured using Pagel’s λ, a scaling parameter of phylogenetic dependence that varies from 0 to 1 (Pagel 1994). A value of λ = 0 means that traits are independent of phylogeny, while λ = 1 indicates that traits are subject to a strong phylogenetic signal. We estimated the λ values using the fitDiscrete function for discrete variables and the phylosig function for continuous variables of the geiger v2.0.7 package [36].

Variation in genome size (the response variable) with respect to the predictor variables (latitude, PCA scores of the bioclimatic variables, microhabitat, and parity mode) and their interaction was assessed using several phylogenetic generalized least-squares models (PGLSs) using the corPagel correlation structure covariance matrix. To assess the contribution of phylogeny, we fitted lambda (λ) in the PGLS and tested each model using λ = 0 and λ = 1 [43]. All models were fitted using the gls function of the nlme package v3.1–158 [44]. Genome size values were log-transformed prior to the analysis and median latitude absolute values were included as a covariate in the models.

The most informative models were selected using the Akaike’s Information Criterion (AIC) implemented in the aictab function of the AICcmodavg v2.3–1 package [45, 46]. Statistical significance of the models was estimated by testing each fitted model against a null model (i.e. a model with all regression parameters equal to 0) using a likelihood ratio test. A significant *P*-value indicated that at least one of the predictor variables in the fitted model had a significant association with the response variable [47]. The *P*-value of each predictor variable included in a statistically significant model was estimated by means of an ANOVA test using the anova function of the stats v4.1.2 package [48]. Post hoc pairwise comparisons were run using the function emmeans of the emmeans v1.7.4–1 package to find significant differences between each pair of estimated marginal means applying the Bonferroni correction [49]. All figures were created using the ggtree v3.3.1, ggnewscale v0.4.6 and ggplot2 v3.3.5 packages and adjusted using InkScape v1.2.2. Statistical analyses were conducted in R v4.1.2 [48] (Additional file 2).

## Results

### Genome size variation across squamates

**Fig. 1:**
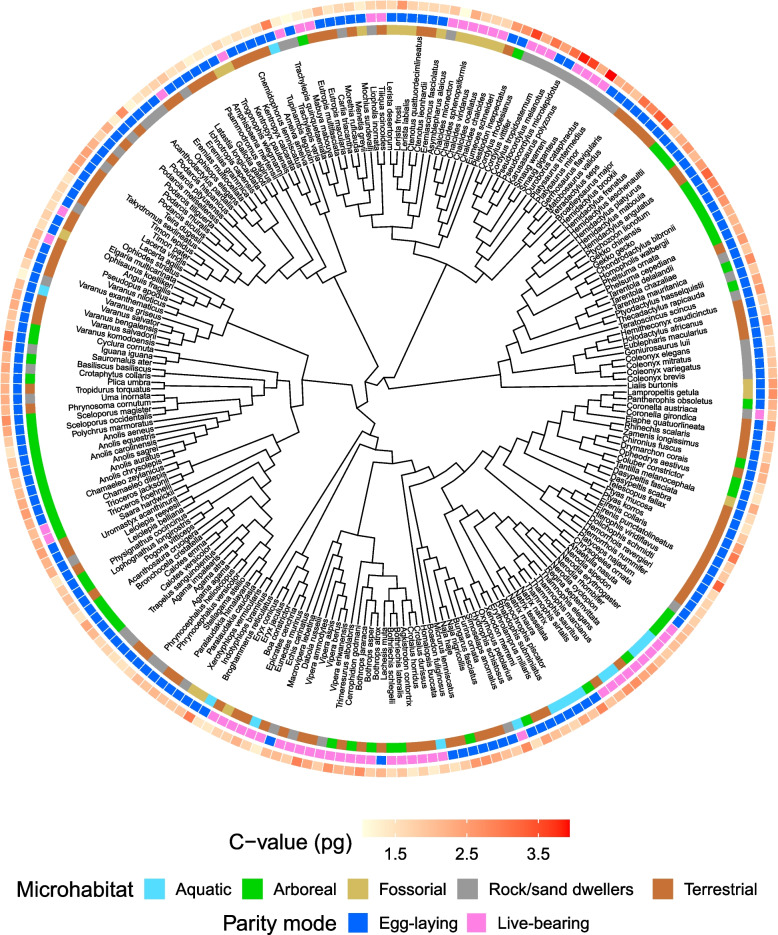
Taxonomy-based dendrogram of squamates with known genome size (*n* = 227). *C*-values are shown in red gradient (minimum/light red = 1.05 pg, maximum/dark red = 3.93 pg). Specific *C*-values are reported in Additional File 1. Differences in microhabitat use and parity mode are also plotted on the dendrogram using different colours

**Fig. 2:**
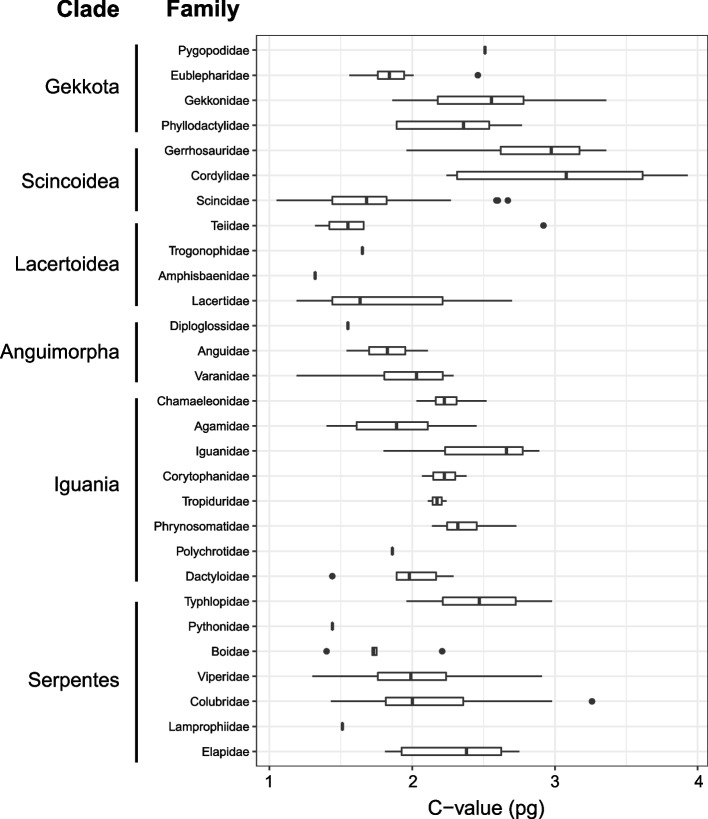
Range of genome size (pg, uncorrected values) for the squamate families included in this study. Where present, the vertical line within the box indicates the median and the horizontal whiskers the 95% confidence intervals

We recorded a 3-fold variation in genome size across 227 species of squamates (Fig. 1). Values ranged from 1.05 pg for the fossorial mionecton skink *Chalcides mionecton* to 3.93 pg for the rock and sand dweller armadillo girdled lizard *Ouroborus cataphractus* (Additional file 1). Genome size values across families showed the highest values for Cordylidae, Gerrhosauridae, Iguanidae, Gekkonidae and Pygopodidae (> 2.5 pg) while the lowest values (< 1.5 pg) were recorded for Pythonidae and Amphisbaenidae (Fig. 2). Arboreal squamates showed higher median values than terrestrial, fossorial, rock/sand dwellers and aquatic species, while larger genomes were found in egg-laying species with respect to live-bearing ones (Fig. S1, Additional file 3).

### Correlation between genome size and predictor variables

The pPCA efficiently reduced the dimensionality of the dataset with the first 2 PC axes explaining 80% of total variation of the species climatic distribution (Table S1, Additional File 3). The first component explained 64.03% of the variance and was mainly positively associated with precipitation (annual precipitation, precipitation in the wettest month and quarter, precipitation in the warmest quarter and driest quarter) and negatively associated with temperature (temperature annual range and temperature seasonality). The second component was negatively associated with rainfall seasonality. A weak phylogenetic signal was recovered for the bioclimatic variables (λ = 0.19).

**Fig. 3:**
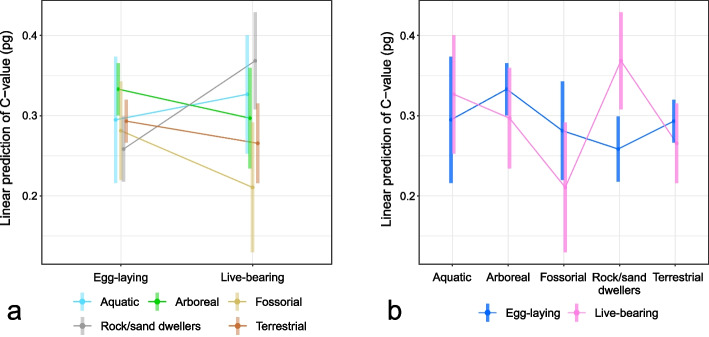
Interaction plot of the phylogenetic generalized least-squares (PGLS) model with the lowest ∆AICc. Each point corresponds to the estimated marginal mean and the bar indicates the standard error with 95% confidence interval for each estimate. Estimated marginal means are compared either by microhabitat use (**a**) or by parity mode (**b**)

Pagel’s λ values indicated a strong phylogenetic signal for all predictor variables (λ > 0.80) while a moderate value was recorded for genome size (λ = 0.48) (Table S2, Additional file 3). The two best-fitting models based on the lowest Akaike’s Information Criterion (∆AICc < 2) did not include phylogeny as a covariate (λ = 0, Table 1). The best-fitting model included microhabitat, parity mode and their interaction (Table 1). The ANOVA test indicated that the interaction between microhabitat and parity mode had a significant effect on genome size (Table 2). The marginal means indicated that larger genome sizes were found in live-bearing species living in rock/sand ecosystems (Emmean = 0.37; SE = 0.03; df = 215; Lower CL = 0.32; Upper CL = 0.43) while lower genome sizes characterized fossorial, live-bearing species (Emmean = 0.22; SE = 0.04; df = 215; Lower CL = 0.14; Upper CL = 0.30) (Fig. 3; Table S3, Additional file 3). Post hoc tests showed that live-bearing species living in rock/sand ecosystems had a significantly larger genome size than egg-laying species living in the same habitat and fossorial, live-bearing species. Arboreal egg-laying species also showed larger genome sizes than egg-laying species living in rock/sand ecosystems (Table S4 and S5, Additional file 3).

**Table 1:** Results of phylogenetic generalized least-squares (PGLSs) models explaining the variation in genome size in squamates as a function of microhabitat, parity mode and bioclimatic variables (PC1, PC2) and their interaction. The number of parameters (K), corrected Akaike’s information criterion (AICc), difference in AICc with respect to the model with the highest support (∆AICc) and Akaike’s weight (*wi*) are given for each model. Absolute value of median latitudes (Lat) and Pagel’s lambda (λ = 0/1) were included as covariates in all models. Asterisks indicate models with *P* < 0.05 after a full-null model comparison

Model	Covariate	K	AICc	∆AICc	*w*_*i*_
Microhabitat × Parity mode*	λ = 0 + Lat	12	− 364.3	0	0.47
PC1	λ = 0 + Lat	4	− 362.04	2.26	0.15
Microhabitat	λ = 0 + Lat	7	− 360.3	4	0.06
Parity mode + PC1	λ = 0 + Lat	5	−359.98	4.32	0.05
Parity mode × PC1	λ = 0 + Lat	6	−359.87	4.43	0.05
PC2	λ = 0 + Lat	4	−359.49	4.81	0.04
Parity mode	λ = 0 + Lat	4	−359.41	4.89	0.04
Microhabitat + PC1	λ = 0 + Lat	8	− 358.79	5.51	0.03
Microhabitat + PC2	λ = 0 + Lat	8	−358.22	6.08	0.02
Microhabitat + Parity mode	λ = 0 + Lat	8	−358.18	6.13	0.02
Parity mode + PC2	λ = 0 + Lat	5	−357.4	6.9	0.01
Parity mode × PC2	λ = 0 + Lat	6	− 356.8	7.5	0.01
Microhabitat + Parity mode + PC1	λ = 0 + Lat	9	− 356.62	7.68	0.01
Microhabitat + Parity mode + PC2	λ = 0 + Lat	9	− 356.09	8.21	0.01
Microhabitat × PC1	λ = 0 + Lat	12	− 355.85	8.45	0.01
Microhabitat × PC2	λ = 0 + Lat	12	− 354.39	9.91	0
Microhabitat × Parity mode × PC2*	λ = 0 + Lat	22	− 353.09	11.21	0
Microhabitat × Parity mode × PC1	λ = 0 + Lat	22	− 349.83	14.47	0
Microhabitat × PC2*	λ = 1 + Lat	12	− 327.3	37	0
PC1	λ = 1 + Lat	4	− 322.27	42.03	0
PC2	λ = 1 + Lat	4	−320.48	43.82	0
Parity mode	λ = 1 + Lat	4	−320.41	43.89	0
Parity mode + PC1	λ = 1 + Lat	5	−320.22	44.08	0
Parity mode × PC1	λ = 1 + Lat	6	−318.56	45.74	0
Parity mode + PC2	λ = 1 + Lat	5	−318.39	45.91	0
Microhabitat	λ = 1 + Lat	7	− 317.78	46.52	0
Microhabitat + PC1	λ = 1 + Lat	8	−317.46	46.84	0
Parity mode × PC2	λ = 1 + Lat	6	−317.43	46.87	0
Microhabitat × PC1	λ = 1 + Lat	12	−316.29	48.01	0
Microhabitat + PC2	λ = 1 + Lat	8	−315.66	48.64	0
Microhabitat + Parity mode	λ = 1 + Lat	8	−315.66	48.64	0
Microhabitat + Parity mode + PC1	λ = 1 + Lat	9	−315.29	49.01	0
Microhabitat + Parity mode + PC2	λ = 1 + Lat	9	− 313.53	50.77	0
Microhabitat × Parity mode × PC2*	λ = 1 + Lat	22	− 313.29	51.01	0
Microhabitat × Parity mode	λ = 1 + Lat	12	− 310.65	53.65	0
Microhabitat × Parity mode × PC1	λ = 1 + Lat	22	− 304.72	59.58	0

**Table 2:** ANOVA result for the best-fitting phylogenetic generalized least-square (PGLS) model (∆AICc = 0). *P*-values < 0.05 in bold

Model	Predictor	df	*F*-value	*P*-value
Microhabitat × Parity mode (λ = 0 + Lat) df = 216	Latitude	1	0.749	0.388
	Microhabitat	4	2.278	0.062
	Parity mode	1	0.341	0.560
	Microhabitat × Parity mode	4	3.669	**0.007**

## Discussion

In this study, we showed that genome size variation in squamates can be explained by interspecific differences in environmental parameters and species parity mode. In particular, live-bearing species living in rock/sand ecosystems and arboreal egg-laying species had larger genome sizes than egg-laying, rock/sand dwellers and fossorial live-bearing species.

Habitat conditions have been found to be among the main factors driving the evolution of genome size in many species [50–52]. Despite patterns of association between environmental factors and genome size often differ across taxa, a general rule has been suggested by Knight et al. [53] known as the large genome constraint hypothesis. The hypothesis postulates that species with large genomes are under-represented in less-stable environments. This seems to be confirmed in many taxa spanning from plants to vertebrates. Plants with large genomes are found in less stressful environments with longer growing seasons [53]. In crustaceans, there is evidence that smaller genome sizes are found in species living in terrestrial environments, which can show a wider range of habitat variation than the marine realm [8, 54]. In vertebrates, salamanders that colonize ephemeral habitats have smaller genomes than species which are strictly bound to permanent and stable aquatic environments [9]. Finally in fish, larger genomes were recorded in deep-sea species living in more stable environments than neritic, epi- or mesopelagic zone dwellers [17].

There are, at the same time, exception to the large genome constraint hypothesis [50]. For instance, some species of freshwater fishes living under relatively unpredictable conditions show larger genome sizes than marine species [6]. Terrestrial molluscs are subject to variable habitat conditions and have larger genomes than aquatic species [55]. Despite large genomes can have high maintenance costs, a larger DNA content might therefore promote genomic stability in unstable environments. Our results on the association between genome size and microhabitat use seems to support a pattern whereby species living in less predictable rock/sand ecosystems show larger genomes than fossorial taxa.

Vinogradov [56] found a positive correlation between genome sizes and GC content in many vertebrate lineages, including squamates. Indeed, GC-rich DNA sequences have high physical stability and are more thermostable than AT-rich sequences [57]. Larger genomes might have been selected in squamates that live in rock and sand habitats to protect their DNA from relatively high temperature and from genetic damage that may derive from higher metabolic rates. Preliminary results by Olmo [58] on large genome sizes and GC content recorded in desert lizards with relatively high body temperatures and tolerance to critical maximum and lethal temperatures seem to be in line with these assumptions. That is consistent with our observation on arboreal species having significantly larger genomes. Species that live in the canopy layer experience higher levels of solar radiation than ground dwellers so that a relatively larger genome might increase resistance to DNA damages caused by ultraviolet light [59, 60]. A similar pattern was observed in birds, whereby arboreal species showed larger genome sizes than species living in open environments [61].

A strong association between developmental complexity and genome size variation has also been described in many studies with a rather homogeneous trend across metazoans. Organisms with complex developments, such as those characterized by a relatively high number of larval stages, have time-limited developmental windows in which to complete either single or successive metamorphoses. This process requires rapid cellular division and differentiation with direct implications on the amount of DNA that can be replicated. On the other hand, time plays a less important role for those species showing an abbreviated or direct development resulting in patterns of genome expansion [62]. This trend is clear in crustaceans, where species with narrow temporal windows between successive larval stages or species with direct development have relatively small genomes (e.g., [8, 63]). In insects, holometabolous species show the most complex developmental mode and have smaller genomes than hemimetabolous taxa [62, 64]. In cyclostomes, anurans, and urodeles, species having a complete metamorphosis present smaller genomes than those with direct development [6, 65]. Live-bearing is a more direct developmental mode than egg-laying and should therefore be associated with larger genome sizes. This is confirmed in chondrichthyans, which are mainly live-bearing species and show larger genomes than ray-finned fishes [6].

According to this hypothesis, live-bearing squamates should present larger genome sizes than egg-laying species. It is however difficult to assess whether larger genomes are specifically linked to one parity mode or the other, for our analyses showed that larger genomes are found in both arboreal egg-laying species and live-bearing species living in rock or sand habitats. This suggests that the correlation between genome size variation and parity mode might also be influenced by habitat type. Indeed, in squamates, both parity mode and embryonic development appears to be influenced by environmental factors [34]. In particular, in cold regions, embryos of live-bearing species develop faster compared to egg-laying embryos [66].

The correlation among habitat, life history traits and genome size variation are also well described in amphibians. In anurans, high temperature and aridity induce an increase in cell replication rate and a contraction of development periods. Similarly, in salamanders, water shortage induces a reduction in pre-metamorphic developmental time. These conditions were also associated to smaller genome sizes [9, 10].

The evolution of genome size in squamates may ultimately be related to cell metabolism, which itself is closely influenced by environmental factors, especially in ectotherms [15]. Cell metabolism and environmental factors are also linked to embryogenesis and parity mode [34] and eventually to clutch size [67], which was found to be correlated with genome size by Chen et al. [19]. These patterns could explain why genome size variation in squamates was mainly correlated to the interaction between parity mode and habitat use in our study.

However, additional variables linked to developmental physiology and cytology, such as species developmental times and cell replication rates should be considered to further investigate the effect of life history traits and the environment on genome size variation in squamates.

In conclusion, ecological factors and parity mode appear to be correlated to genome size variation in squamate reptiles. Our results suggest that genome size may favour adaptation of some species to certain environments (e.g. allow for higher resistance to ultraviolet radiation) or could otherwise result from the interaction between environmental factors and parity mode. A strong relationship, with causation proceeding in both directions, seems to occur between genome size and the phenotype of an organism, with several ecological and physiological factors exerting synergistic effects on this interaction [62]. Whether genome size and structure are a consequence of environmental factors or favour adaptation of species to new habitats is yet to be clarified.

Non-adaptive forces involving genome content modelling, which we did not consider in this study, have often been indicated as factors possibly involved in the evolution of genome size. In many animals and plants, substantial variation in genome size between species was suggested to be the result of differential rates of transposable element accumulation [1, 68, 69]. This led to the prevailing view that genomic repeat abundance and genome size tend to tightly co-evolve [70]. Squamates, however, seem not to be consistent with this hypothesis. In fact, despite the extreme variation in genomic repeat element content, genome size across this lineage is remarkably conserved, suggesting the presence of a dynamic equilibrium in which genomic DNA gain through transposable elements expansion may be balanced by genomic DNA loss through deletion [71]. Future research may therefore integrate information on genome size, ecological factors and life history traits with genome sequencing data in order to understand the possible role of differential genome content in the evolutionary process of genome size variation in squamates.

### Supplementary Information

**Additional file 1. **Dataset of 227 squamate species with known *C*-values. Ecological and parity mode data are reported for each species.

**Additional file 2.** R code used in this study.

**Additional file 3:** **Supplementary results.** Fig. S1, Table S1, S2, S3, S4 and S5.

## Data Availability

The dataset supporting the conclusions of this article is included within the article and its additional files.

## Abbreviations

DNA: Deoxyribonucleic Acid
GoaT: Genomes on a Tree
pg: picograms
GBIF: Global biodiversity information facility
pPCA: Phylogenetic principal components analysis
PCA: Principal component analysis
PC: Principal component
PGLS: Phylogenetic generalized least-squares
AIC: Akaike’s information criterion
ANOVA: Analysis of variance
PC1: First principal component
PC2: Second principal component
GC: Guanine and cytosine
AT: Adenine and thymine
