# Supplementary material for: Ecological factors and parity mode correlate with genome size variation in squamate reptiles
Source: BMC Ecol Evol. 2023 Dec 5;23:69. doi: 10.1186/s12862-023-02180-4 (PMC10696768; doi:10.1186/s12862-023-02180-4)
Supplement: Supplementary file 3 — Additional file 3: Supplementary results. Fig. S1, Table S1, S2, S3, S4 and S5. [file 12862_2023_2180_MOESM3_ESM.docx]

***Supplemental information***

*for:*

**Ecological factors and parity mode correlate with genome size variation in squamate reptiles**

**Anik Saha^1^, Arianna Bellucci^1^, Sara Fratini^1^, Stefano Cannicci^1,2^, Claudio Ciofi^1,*^, Alessio Iannucci^1,2,*^**

^1^Department of Biology, University of Florence, Sesto Fiorentino, Italy

^2^NBFC, National Biodiversity Future Center, Palermo 90133, Italy

*** Correspondence:**

Alessio Iannucci, email: [alessio.iannucci@unifi.it](mailto:alessio.iannucci@unifi.it); Claudio Ciofi, email: claudio.ciofi@unifi.it.

**Figure S1.** Genome size (pg, uncorrected values) in species of squamates grouped according to microhabitat use (a) and parity mode (b). The boxplots whiskers indicate the 95% confidence intervals and the dots indicate the outliers.

**
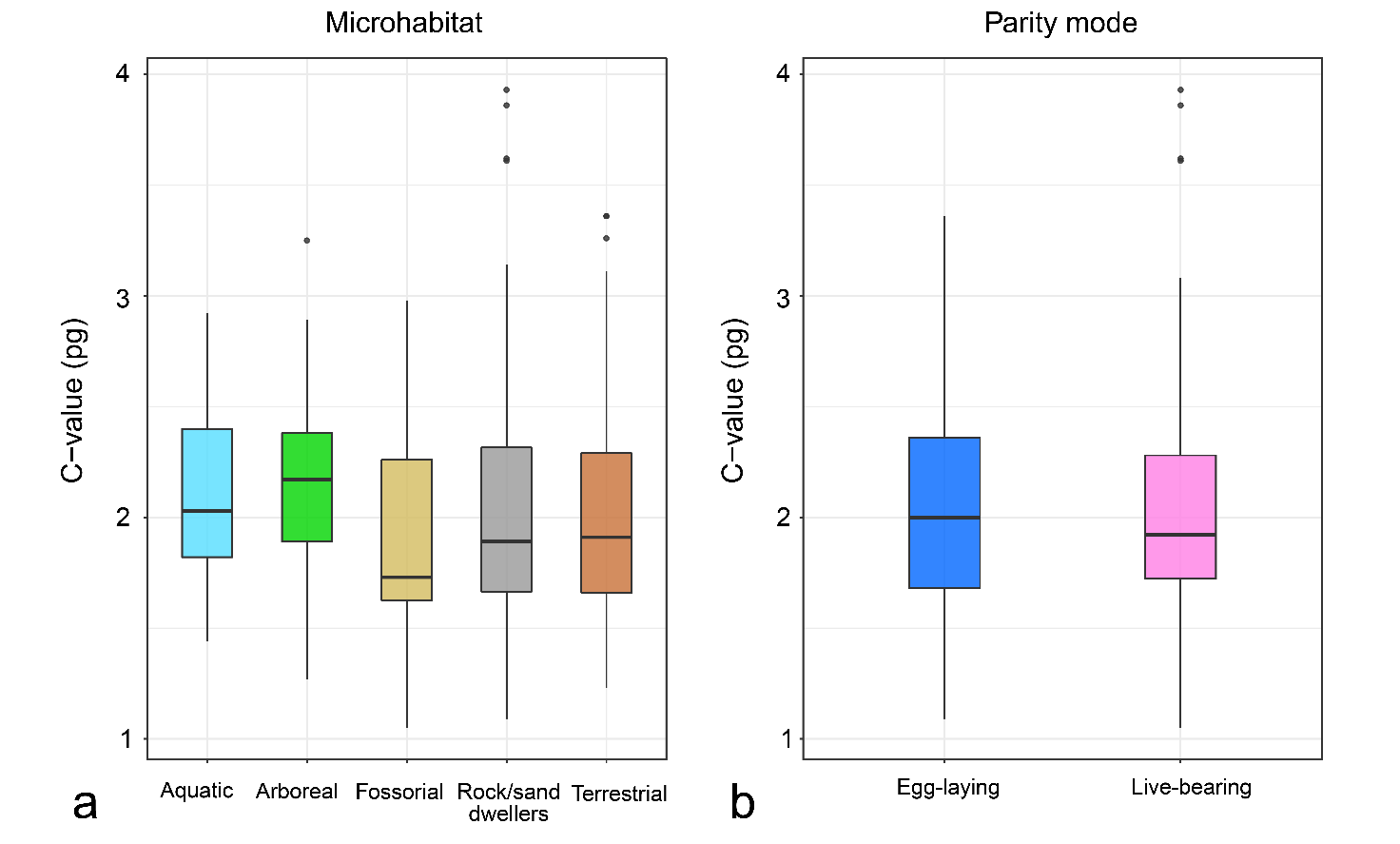
**

**Table S1.** Loadings of the first four components of the phylogenetically corrected Principal Component Analysis (pPCA). Loadings are given along with their proportion of variance. Values of loading ≥ 0.75 are highlighted in bold.

| **Climatic variables** | **PC1** | **PC2** | **PC3** | **PC4** |
| --- | --- | --- | --- | --- |
| Bio1 = Annual mean temperature | 0.52 | -0.54 | 0.32 | -0.20 |
| Bio2 = Mean diurnal range | -0.52 | -0.07 | -0.18 | -0.21 |
| Bio3 = Isothermality | 0.66 | -0.36 | 0.38 | -0.45 |
| Bio4 = Temperature seasonality | **-0.77** | 0.31 | -0.41 | 0.33 |
| Bio5 = Max temperature of warmest month | -0.13 | -0.33 | 0.13 | -0.08 |
| Bio6 = Min temperature of coldest month | 0.56 | -0.35 | 0.44 | -0.16 |
| Bio7 = Temperature annual range | **-0.79** | 0.29 | -0.39 | 0.20 |
| Bio8 = Mean temperature of wettest quarter | 0.52 | -0.48 | -0.22 | -0.28 |
| Bio9 = Mean temperature of driest quarter | 0.31 | -0.28 | 0.62 | -0.02 |
| Bio10 = Mean temperature of warmest quarter | 0.14 | -0.40 | 0.15 | -0.07 |
| Bio11 = Mean temperature of coldest quarter | 0.60 | -0.49 | 0.40 | -0.22 |
| Bio12 = Annual precipitation | **0.97** | -0.05 | -0.06 | 0.20 |
| Bio13 = Precipitation of wettest month | **0.92** | -0.31 | 0.00 | 0.22 |
| Bio14 = Precipitation of driest month | 0.72 | 0.60 | -0.18 | -0.22 |
| Bio15 = Precipitation seasonality | -0.13 | **-0.85** | 0.21 | 0.27 |
| Bio16 = Precipitation of wettest quarter | **0.92** | -0.27 | -0.02 | 0.26 |
| Bio17 = Precipitation of driest quarter | **0.76** | 0.59 | -0.20 | -0.17 |
| Bio18 = Precipitation of warmest quarter | **0.85** | -0.16 | -0.49 | -0.09 |
| Bio19 = Precipitation of coldest quarter | 0.65 | 0.57 | 0.46 | 0.18 |
|  |  |  |  |  |
| Standard deviation | 0.06 | 0.03 | 0.02 | 0.01 |
| Proportion of variance | 0.64 | 0.16 | 0.11 | 0.05 |
| Cumulative proportion | 0.64 | 0.80 | 0.91 | 0.96 |

**Table S2.** Values of phylogenetic signal, estimated as Pagel’s lambda (λ), for the response and the predictor variables. For each variable, the log-likelihood (LL) is reported.

| **Variable** | **Pagel’s lambda (λ)** | **LL** |
| --- | --- | --- |
| C-value | 0.48 | 209.63 |
| Latitude | 0.92 | -883.17 |
| Microhabitat | 0.81 | -284.34 |
| Parity mode | 1.00 | -81.50 |

**Table S3.** Marginal means of the phylogenetic generalized least-squares (PGLS) model with the lowest ∆AICc. Standard error (SE), degree of freedom (df) and lower and upper confidence limits (Lower CL and Upper CL) are shown.

| **Predictor** | **Emmean** | **SE** | **df** | **Lower CL** | **Upper CL** |
| --- | --- | --- | --- | --- | --- |
| Aquatic egg-laying | 0.30 | 0.04 | 215 | 0.22 | 0.38 |
| Arboreal egg-laying | 0.34 | 0.02 | 215 | 0.31 | 0.37 |
| Fossorial egg-laying | 0.29 | 0.03 | 215 | 0.23 | 0.35 |
| Rock/sand dwellers egg-laying | 0.26 | 0.02 | 215 | 0.23 | 0.30 |
| Terrestrial egg-laying | 0.30 | 0.01 | 215 | 0.27 | 0.32 |
| Aquatic live-bearing | 0.33 | 0.04 | 215 | 0.26 | 0.41 |
| Arboreal live-bearing | 0.30 | 0.03 | 215 | 0.24 | 0.37 |
| Fossorial live-bearing | 0.22 | 0.04 | 215 | 0.14 | 0.30 |
| Rock/sand dwellers live-bearing | 0.37 | 0.03 | 215 | 0.32 | 0.43 |
| Terrestrial live-bearing | 0.27 | 0.02 | 215 | 0.22 | 0.32 |

**Table S4.** *Post hoc* test for the significant predictor (Microhabitat × Parity mode) included in the phylogenetic generalized least-squares (PGLS) model with the lowest ∆AICc (contrast by microhabitat). Bonferroni correction with a 95% confidence interval was applied. *P*-values < 0.05 are highlighted in bold.

| **Contrast** | **Estimate** | **SE** | **df** | **t-ratio** | ***P*-value** |
| --- | --- | --- | --- | --- | --- |
| Aquatic live-bearing - Aquatic egg-laying | 0.032 | 0.054 | 215 | 0.584 | 0.560 |
| Arboreal live-bearing - Arboreal egg-laying | -0.036 | 0.036 | 215 | -1.015 | 0.311 |
| Fossorial live-bearing - Fossorial egg-laying | -0.071 | 0.050 | 215 | -1.410 | 0.160 |
| Rock/sand dwellers live-bearing - Rock/sand dwellers egg-laying | 0.110 | 0.034 | 215 | 3.211 | **0.002** |
| Terrestrial live-bearing - Terrestrial egg-laying | -0.028 | 0.027 | 215 | -1.019 | 0.309 |

**Table S5.** *Post hoc* test for the significant predictor (Microhabitat × Parity mode) included in the phylogenetic generalized least-squares (PGLS) model with the lowest ∆AICc (contrast by parity mode). Bonferroni correction with a 95% confidence interval was applied. *P*-values < 0.05 are highlighted in bold.

| **Contrast** | **Estimate** | **SE** | **df** | **t-ratio** | ***P*-value** |
| --- | --- | --- | --- | --- | --- |
| Egg-laying arboreal - Egg-laying aquatic | 0.038 | 0.043 | 215 | 0.889 | 1.000 |
| Egg-laying fossorial - Egg-laying aquatic | -0.014 | 0.050 | 215 | -0.273 | 1.000 |
| Egg-laying fossorial - Egg-laying arboreal | -0.052 | 0.035 | 215 | -1.497 | 1.000 |
| Egg-laying rock/sand dwellers - Egg-laying aquatic | -0.036 | 0.044 | 215 | -0.827 | 1.000 |
| Egg-laying rock/sand dwellers - Egg-laying arboreal | -0.075 | 0.025 | 215 | -2.957 | **0.035** |
| Egg-laying rock/sand dwellers - Egg-laying fossorial | -0.023 | 0.035 | 215 | -0.644 | 1.000 |
| Egg-laying terrestrial - Egg-laying aquatic | -0.002 | 0.042 | 215 | -0.042 | 1.000 |
| Egg-laying terrestrial - Egg-laying arboreal | -0.040 | 0.021 | 215 | -1.946 | 0.530 |
| Egg-laying terrestrial - Egg-laying fossorial | 0.012 | 0.033 | 215 | 0.365 | 1.000 |
| Egg-laying terrestrial - Egg-laying rock/sand dwellers | 0.035 | 0.022 | 215 | 1.567 | 1.000 |
| Live-bearing arboreal - Live-bearing aquatic | -0.02978 | 0.049 | 215 | -0.608 | 1 |
| Live-bearing fossorial - Live-bearing aquatic | -0.11608 | 0.0547 | 215 | -2.123 | 0.349 |
| Live-bearing fossorial - Live-bearing arboreal | -0.0863 | 0.0516 | 215 | -1.673 | 0.9578 |
| Live-bearing rock/sand dwellers - Live-bearing aquatic | 0.04183 | 0.0475 | 215 | 0.881 | 1 |
| Live-bearing rock/sand dwellers - Live-bearing arboreal | 0.07161 | 0.0438 | 215 | 1.635 | 1 |

**Table S5.** *Continued.*

| Live-bearing rock/sand dwellers - Live-bearing fossorial | 0.15791 | 0.0493 | 215 | 3.204 | **0.0156** |
| --- | --- | --- | --- | --- | --- |
| Live-bearing terrestrial - Live-bearing aquatic | -0.0611 | 0.0444 | 215 | -1.377 | 1 |
| Live-bearing terrestrial - Live-bearing arboreal | -0.03132 | 0.0403 | 215 | -0.778 | 1 |
| Live-bearing terrestrial - Live-bearing fossorial | 0.05499 | 0.0466 | 215 | 1.181 | 1 |
| Live-bearing terrestrial - Live-bearing rock/sand dwellers | -0.10292 | 0.0379 | 215 | -2.716 | 0.0715 |
